# Investigating the types of microorganisms causing cerebrospinal fluid shunt infection in King Abdullah University Hospital in Jordan

**DOI:** 10.5339/qmj.2025.11

**Published:** 2025-02-23

**Authors:** Sulieman Daoud, Atef Hulliel, Sultan Jarrar, Amer Jaradat, Mohammad Jamous, MajdM. Al Barakat, Aseel Rabadi, Nataly Al-zu'bi

**Affiliations:** ^1^Neurosurgery Department, Irbid, Jordan; ^2^Faculty of Medicine, Jordan University of Science & Technology, Irbid, Jordan *Correspondence: Sulieman Daoud. Email: ssdaoud@just.edu.jo

**Keywords:** Ventriculoperitoneal shunt, *Acinetobacter baumannii*, coagulase-negative staphylococci

## Abstract

**Background:**

Ventriculoperitoneal shunt (VPS) placement treats hydrocephalus by draining excess cerebrospinal fluid (CSF). Despite advances, infections remain a common complication, resulting in significant morbidity and mortality. Infection rates range from 7.2 to 18%, with common pathogens being *Staphylococcus epidermidis* and *Staphylococcus aureus*. Risk factors include young age, postoperative CSF leakage, prolonged surgery, and previous infections. The aim of this study was to describe the prevalence of CSF shunt infections at King Abdullah University Hospital (KAUH), assess infection rates in pediatric and adult patients, and report causative microorganisms.

**Methods:**

A retrospective analysis was conducted on all patients with CSF shunt-related infections in our hospital (KAUH) over the last 17 years (2005–2023). The patients’ demographics, laboratory results, and details of causative microorganisms were collected.

**Results:**

Of the 579 patients who underwent CSF shunting at KAUH in Jordan, 59 (10.1%) had a positive CSF culture for shunt infection. The majority of the patients were children (83.1%) with a median age of 9 months and a higher proportion of male patients (57.6%). Most of the infections were due to congenital anomalies (74.6%). The median time to infection was 13 days, with 13.5% experiencing recurrent infections. Recurrent infection rates were found to be significantly higher in pediatric patients (*p* = 0.00007). The most common pathogens were *Acinetobacter baumannii* (47.5%) and *Staphylococcus* species (40.7%). Analysis by age group showed a significant association between age and *Acinetobacter baumannii* infections (*p* = 0.008).

**Conclusion:**

The study provided demographic and microbiological data on VPS infections, with *Acinetobacter baumannii* being the most common causative organism. Treatment of these infections remains challenging, highlighting the need for more comparative research on different treatment options.

## Introduction

Ventriculoperitoneal shunt (VPS) placement is the most common neurosurgical procedure used to treat hydrocephalus, a condition characterized by an accumulation of cerebrospinal fluid (CSF) in the ventricles of the brain.^1^ Despite major advances in surgical procedures, sterility, and equipment that enhanced its effectiveness, VPS insertion carries a risk of complications, with infections being the most common and challenging to manage.^1^

Shunt infections are considered the second most common cause of shunt malfunction. In the past, several studies reported that the incidence of shunt infections ranged between 7.9 and 18%.^2–5^ More recent studies indicate that infection rates have shown similar findings. For example, a 2023 study reported an incidence rate of 13.9%,^6^ while a more recent study from 2024 reported a lower incidence rate of 7.2%.^7^ Shunt infection poses significant risks of morbidity and mortality, with associated complications such as seizures, increased likelihood of future shunt infections, and impaired school performance in children.^8^ *Staphylococcus epidermidis*, *Staphylococcus aureus*, and gram-negative rods are the most commonly implicated microorganisms in CSF shunt infections.^6,9–11^

Numerous studies have identified several risk factors for shunt infections, which often interact in complex ways. For example, young age is consistently associated with an increased risk of shunt infection, as well as postoperative CSF leakage.^4^ Increased manual contact with the shunt system during the procedure can further increase the risk of infection,^12^ as well as result in prolonged surgery time^12^ and glove perforations during shunt manipulation.^4^ African American ethnicity,^13^ public insurance,^13^ previous shunt infections,^9^ and the cause of intraventricular hemorrhage^13^ are also associated with higher infection rates.

Premature neonates, whose immune systems are not yet fully developed, have the highest reported risk of shunt infections.^5,9,14^ However, some studies have shown that implementing strict preventive measures reduces shunt infection rates. For example, one study reported a shunt infection rate of only 1.33% when a uniform surgical technique, limited manipulation of the shunt system and skin edges, and double gloving were introduced in all procedures.^8^

To achieve these objectives, we conducted a retrospective study at our institute, King Abdullah University Hospital (KAUH) in Jordan, with the aim of determining the incidence of infections resulting from CSF shunt procedures and the microorganisms responsible for them, with a brief review of the prevention methods and the treatment plan.

## Materials And Methods

### Patients

We collected medical records of all patients diagnosed with VPS infections who were treated at the Neurosurgery Department in KAUH over the last 17 years (2005–2023). Shunt infections were defined as the presence of pathogenic organisms in CSF cultures. Therefore, only cases with positive CSF cultures were included, regardless of the white blood cell (WBC) count (elevated or not) in the CSF. For recurrent infections, only data on the primary infection were included. Patient data were retrieved through a search of the hospital's electronic medical record (EMR) system using specific keywords and diagnostic codes to identify cases of shunt infection. This study was approved by the Institutional Review Board Committee at KAUH (IRB no. 101/132/2020).

### Data extraction

Information extracted included patients’ age, gender, indications for insertion, dates of both insertion and shunt infection, culture results and types of organisms, and whether or not the patient experienced recurrent infections.

### Statistical analysis

Statistical analysis was conducted using IBM SPSS Statistics v26.0. Descriptive measures included counts and proportions for categorical data (%) and medians with IQR (interquartile range) for continuous data. For the comparison of infectious microorganisms, patients were stratified according to different age groups (pediatrics ( < 18 years) vs. adults ( ≥ 18 years)). Chi-square tests, or Fisher's exact tests (when one cell count was ≤ 5), were used as tests of association. The pairwise deletion method was used for missing data that were assumed to be completely missing at random. While this method helps to make use of available data, it assumes that data are missing completely at random and could introduce potential bias if this assumption is not met. A two-sided *p* value of ≤ 0.05 was considered statistically significant.

## Objectives

The aim of this study was to describe the epidemiological data on CSF shunt infections at KAUH, including the prevalence of infections in pediatric and adult patients and the rate of recurrent infections. The study also aimed to report the frequency and type of causative microorganisms, with a particular focus on *Acinetobacter baumannii* and *Staphylococcus* species, and to analyze the association between specific microorganisms and patient age groups.

## Results

### Epidemiology

Of the total 579 patients who underwent a CSF shunting procedure at KAUH, 59 (10.1%) had a positive CSF culture for shunt infection. The patients included were mostly from the pediatric group (83.1%, 49/59), with a median age of 9 months (range: 1 day–72 years) and a male predominance of 57.6% (34/59). The CSF shunting procedure was mostly indicated due to congenital anomalies (74.6%). The median time from insertion surgery to infection was 13 days (range: 0 days–3 years). Recurrent infections were found in 13.5% (8/59) of patients with available data. The rate was significantly higher in the pediatric group than in the adult group (*p* = 0.00007). The patients’ demographic data and laboratory results are summarized in Table 1.

### Infective microorganisms

Overall, most infections were caused by *Acinetobacter baumannii* spp. (47.5%, 28/59), with *Staphylococcus* spp. being the second most common (40.7%, 24/59). After separating the microorganisms by age group (above 18 years and below 18 years), a Fisher's exact test was performed. The results showed a significant *p* value of 0.008, indicating a strong and statistically important association between age and *Acinetobacter baumannii* causing VPS infections (Table 2).

## Discussion

Infection, as discussed previously, is the second most common complication of CSF shunt procedures, with a mortality rate of 10.1%.^15^ An early study reported a low shunt infection rate of 2.6%. However, this low percentage was not due to an actual reduction in the infection rate, but rather due to poor detection and diagnosis.^16^ Therefore, shunt infection is a feared condition because it increases the mortality rate from 17 to 40%.^17^ The varying shunt infection rates (5–27%) highlight the need for effective infection prevention strategies as well as early detection and management of shunt infections.^18,19^ The exact determinants of CSF shunt infection risk are not fully elucidated. However, previous research has identified several risk factors such as patient gender, age at surgery, history of shunt infection, and indication for CSF shunt implantation.^19^ Regarding VPS insertion, numerous studies have shown varying degrees of association between certain etiological factors of hydrocephalus and the risk of VPS infection. Consistent with these findings, the predominant diagnosis leading to CSF shunt insertion in our study was congenital malformation (hydrocephalus), which accounted for 74.6% of cases. At our institution, we defined shunt infection as the presence of pathogenic organisms in CSF cultures. Therefore, only cases with positive CSF cultures were included, regardless of the WBC count (elevated or not) in the CSF. This approach is consistent with previous studies in which infections were characterized by detecting microbial growth in the CSF. Other methods used in previous studies include observation of CSF pleocytosis or low CSF glucose levels in association with clinical features of central nervous system infection.^20^ At our hospital, a uniform set of procedures for shunt insertion was implemented by various surgeons. These measures include administering perioperative antibiotics, shaving, double gloving, minimizing operation time, reducing operating room traffic, and using the same hardware device.

The rate of CSF shunt infections in our study was 10.1%, which is in the range of shunt infections reported in other studies. When the literature was reviewed according to causative agents, it was found that *Acinetobacter* spp. are the leading cause of shunt-related infections, with *Acinetobacter baumannii* identified as the most prevalent pathogen responsible for 47.5% of cases. After *Acinetobacter*, *Staphylococcus* spp. and gram-negative bacteria were reported as the second most common pathogens.^21^ The results of our study showed that the majority of VPS infections were caused by *Acinetobacter* spp., which is rarely found. This rate is higher than typically reported, possibly due to the high resistance of *Acinetobacter* to commonly used antibiotics such as colistin. Several studies have emphasized the growing antibiotic resistance in *Acinetobacter* spp.^22,23^ The high prevalence of *Acinetobacter* spp. in our study may also be attributed to its ability to form biofilms on medical devices such as CSF shunts. Biofilm formation in *Acinetobacter baumannii* acts as an effective protective mechanism that not only contributes to antimicrobial resistance but also enhances the bacteria's ability to survive in hospital environments, particularly on biotic and abiotic surfaces. This biofilm-associated resistance complicates treatment and contributes to the persistence and spread of infections in healthcare settings.^24^ The global prevalence of biofilm-producing *Acinetobacter baumannii* has been reported to reach up to 65.63%, with significant differences observed across different continents. One published meta-analysis shows that Asia has some of the highest prevalence rates, followed by Africa. These geographical differences could be influenced by different healthcare practices, infection control protocols, and the presence of multidrug-resistant strains in different regions.^24^ When comparing infection rates between children and adults in our study, the infection rate in children ( < 18 years) was 17% compared to adults, which was 3%, with a significant difference. Many studies showed that age at initial shunt insertion correlated with a higher risk of infection.^25,26^

When stratifying the most common microorganisms for each age group, infections due to *Acinetobacter* spp. were most common in pediatric age groups, while *Staphylococcus* spp. were most common in adult age groups.

These findings are clinically important given the high resistance rate of *Acinetobacter* to the most commonly used antibiotics, which could serve as a guide for the empirical antibiotics for a better outcome.

However, it is important to continue treatment for Gram-positives such as coagulase-negative staphylococci, as it is still considered as the most common microorganism in numerous studies.^21^

Due to the lack of controlled clinical studies, it is challenging to define optimal treatment practices for CSF shunt infections. Although wide variations are reported, the most conservative approach involves the use of appropriate antibiotics and removal of the infected shunt with the insertion of a temporary external ventricular drainage (EVD) until the CSF becomes sterile.

The protocol used in our department to treat shunt infections was to remove the hardware, insert an EVD, and administer intravenous antibiotic treatment according to the sensitivity of the cultures until we obtained three consecutive negative cultures for at least 14 days. Not to mention, we were always in direct contact with Infectious disease (ID) specialists to adjust the type and dosage of the antibiotic. For resistant cases, we considered intrathecal antibiotics as recommended by the ID specialist.

Some patients experience multiple CSF shunt reinfections. A study conducted by Erps et al. reported that the most significant risk factor for reinfection was a previous shunt infection within the last 6 months.^26^ A study that included 31 children with a second shunt infection found that patients who developed a second shunt infection had a significantly higher risk of subsequent reinfection.^27^ In our study, the reinfection rate was 13.5%.Our study had several methodological limitations. The retrospective nature of the study and inconsistencies in historical record-keeping may have led to incomplete or biased data collection. Additionally, the relatively small sample size limited the statistical power of our analyses and limited the use of more advanced statistical methods. These limitations may have affected the robustness and generalizability of our findings. Future research with prospective data collection and larger sample sizes would allow for more comprehensive analyses and more meaningful conclusions. Additionally, studying the effectiveness of targeted antibiotic therapies against resistant pathogens such as *Acinetobacter baumannii* could provide valuable insights into improving treatment outcomes. Regular monitoring of antibiotic resistance patterns in *Acinetobacter* spp. at our institution will also help inform treatment decisions. Despite these limitations, our study provides valuable insights into the epidemiological and microbiological aspects of CSF shunt infections.

## Conclusion

In our study, we described demographic data and details of the infective microorganisms related to CSF shunt infections at KAUH, with *Acinetobacter baumannii* being the most common causative organism associated with VPS infection. Although focusing on a single institution was necessary due to limited access to data from other centers, this means our findings may not fully represent infection patterns elsewhere. Management of CSF shunt infections remains challenging, especially due to the lack of randomized clinical trials. Therefore, we emphasize the need for further comparative research on different treatment modalities.

### Competing interests

The authors have no conflicts of interest to declare.

### Authors’ contributions

**S.D.**: Conception and design of the study, manuscript drafting, final approval. **S.J.**: Conception and design of the study, data acquisition, final approval. **A.J.**: Data analysis and interpretation, final approval. **M.J.**: Conception and design of the study, final approval. **A.H.**, **M.A.**: Data acquisition, analysis, and interpretation, manuscript revision, final approval. **N.A.**, **A.R.**: Data acquisition, manuscript revision, final approval. All authors read and approved the final manuscript.

## Figures and Tables

**Table 1.** Patients’ demographic data and laboratory results (*n* = 59).

Age, pediatrics (*n*, %)	49 (83.1)

Gender, male (*n*, %)	34 (57.6)

Indications for insertion (*n*, %), *n* = 34	

Congenital	44 (74.6)

Tumor	5 (8.5)

Posterior brain hemorrhage	2 (3.4)

Trauma	1 (1.7)

NPH	3 (5.1)

Others	2 (6.7)

Time from insertion to infection, days (median)Recurrent infection (*n*, %), *n* = 59	138 (13.5)


NPH: normal pressure hydrocephalus.

**Table 2.** Infective microorganisms stratified by age group (*n* = 59).

Microorganisms	< 18 years (*n*, %)	>18 years (*n*, %)

Acinetobacter sp.	25 (51.02%)	3 (30.00%)

Staphylococcus sp.	19 (38.78%)	5 (50.00%)

Candida sp.	1 (2.04%)	0 (0.00%)

Enterococcus sp.	2 (4.08%)	0 (0.00%)

Haemophilus sp.	0 (0.00%)	1 (10.00%)

Klebsiella sp.	0 (0.00%)	1 (10.00%)

Streptococcus sp.	2 (4.08%)	0 (0.00%)

